# Psychosocial Distress and Quality of Life Among Patients With a Chronic Skin Disorder at a Tertiary Care Hospital in Eastern India: A Hospital-Based Case-Control Study

**DOI:** 10.7759/cureus.29830

**Published:** 2022-10-02

**Authors:** Pratyush R Behera, Sarika Palepu, Chandra S Sirka, Rajeev Ranjan, Swetalina Pradhan, Arvind K Singh

**Affiliations:** 1 Medicine, All India Institute of Medical Sciences, Bhubaneswar, IND; 2 Community Medicine and Family Medicine, All India Institute of Medical Sciences, Kalyani, IND; 3 Dermatology, All India Institute of Medical Sciences, Bhubaneswar, IND; 4 Department of Psychiatry, All India Institute of Medical Sciences, Patna, IND; 5 Dermatology, All India Institute of Medical Sciences, Patna, IND; 6 Community Medicine and Family Medicine, All India Institute of Medical Sciences, Bhubaneswar, IND

**Keywords:** anxiety, depression, psychological distress, quality of life, skin disorder

## Abstract

Background

Chronic skin disorder affects the physical and psychological well-being of patients. The impact of psychological burden ranges from low self-esteem and stress to anxiety and depression. Hence, this study was conducted to compare the psychological distress and quality of life (QoL) of patients with a comparative group without any apparent skin disorders.

Methods

This hospital-based case-control study was done on adult patients (≥18 years) suffering from any chronic skin disorder for three or more months and attending the dermatology out patient department (OPD) of a tertiary care institution of Eastern India. Data was collected from 101 patients and 101 controls (matched for age, gender, and place of residence) after obtaining written informed consent in May and June, 2017. Depression was assessed using the Patient Health Questionnaire-9, anxiety was assessed using the Generalised anxiety disorder-7 scale, and health-related quality of life (HRQoL) was assessed using the World Health Organization Quality-of-Life Scale (WHOQoL-BREF). Prevalence and mean scores were compared using Chi-square test and t-test.

Results

About half (49.5%) of the cases had clinically significant impairment of their dermatological quality of life. Clinically significant depression and anxiety was found in 45.54% and 41.58% patients respectively. Depression (OR=4.13, 95% CI 2.06-8.45) and anxiety (OR=4.42, CI=2.13-9.51) were significantly higher in cases as compared to the controls. No significant difference was seen in HRQoL scores.

Conclusion

Screening for anxiety, depression, and QoL should be done for patients of chronic skin disorders attending dermatology OPD so that appropriate psychiatric consultation can be offered to those in need.

## Introduction

The prevalence of both psychological and skin conditions is ever increasing due to changing lifestyle, environmental and occupational factors in industrializing countries like India [1,2]. Skin disorders, though not associated much with mortality and long-term disability, are a great cause of discomfort, pain, mild to moderate physical disability, social stigmatization, and psychological stress. It has been found that the prevalence of depression and anxiety is higher in patients with skin disorders [3]. Chronic skin disorders (CSD) also lead to an impaired quality of Life (QoL) [4]. Psychological distress and impaired QoL are found to be more alarming when the skin lesion is on any of the exposed or visible parts of the body [5]. It is noteworthy that psychological distress leads to exacerbation of skin conditions as such and also leads to resistance to a regular course of treatment [6]. 

Studies from India have focused mostly on specific skin disorders like psoriasis and vitiligo [7-9]. These conditions mostly manifest in severe form and do not represent all kinds of skin disorders causing psychological distress. It would be imperative to assess all CSD that have a visible impact rather than only serious and debilitating conditions. 

Hence, the present study was planned to assess psychological distress and QoL among patients presenting with CSD at a dermatology outpatient department (OPD) of a medical college if Eastern India and also to compare with a control group without any visible skin disorders. We also aimed to explore the disease and patient-related factors so that targeted psychological intervention can be recommended.

## Materials and methods

Study design

The present hospital-based case-control study was done in the dermatology OPD of All India Institute of Medical Sciences (AIIMS), Bhubaneswar, India. The dermatology OPD is functional six days a week and caters services to about 150 patients on every functional OPD day.

Sample size calculation

Assuming the prevalence of depression or anxiety as 20% in controls and 39.4% in the cases [7] and taking alpha as 0.05, power as 80%, and non-response rate of 10%, the sample size in each group was calculated as 101.

Inclusion criteria

Adult patients (≥18 years) attending the dermatology OPD, diagnosed by a dermatologist as suffering from any chronic skin disorder (≥3 months), not having any serious, debilitating condition or communication problems, and provided consent were included. Controls matched by age, sex, and place of residence (urban/rural) were included.

Exclusion criteria

Patients who could not understand the interview questions in any of the three languages (English, Hindi, and Odia) were excluded from the study.

Assessment tools

Depression was assessed using Patient Health Questionnaire-9 (PHQ-9) [10]. A PHQ score of <10 was considered as none or mild depression, and ≥10 was considered as moderate to severe depression.

Anxiety was assessed using the Generalised Anxiety Disorder-7 (GAD-7) scale [11]. A GAD score of <10 was considered as none or mild anxiety, and ≥10 was considered as moderate to severe anxiety.

Dermatological QoL was assessed using the Dermatological Life Quality Index (DLQI) [12]. A DLQI score of ≤10 was considered as none/mild/moderate and >10 as large to extremely large impairment. This scale was administered only to patients with CSD.

Health-related QoL (HRQoL) was assessed using the World Health Organization Quality-of-Life Scale (WHOQoL-BREF), consisting of four domains. The scores are scaled in a positive direction (i.e., higher scores denote a higher quality of life). Within each domain, mean scores were calculated and then multiplied by four in order to make domain scores comparable with the World Health Organization Quality of Life - 100 questions (WHOQOL-100). The scores were subsequently transformed to a 0-100 scale, using the formula (score-4) x (100/16).

Severity scales like Psoriasis Area and Severity Index, Vitiligo Area and Severity Index, Global Acne Grading System, and Scoring Atopic Dermatitis (SCORAD) were used.

If no scale was available to measure the severity of a particular disease, involvement of more than 10% of body surface area was considered severe.

Operational definitions

In this study, chronic skin disorder was defined as any dermatological condition persisting beyond three months of duration, with or without clinical management.

Questionnaire design and validation

Odia and Hindi language translations of all the questionnaires and back translation to English were done by two independent persons not involved in the study. The principal investigator of the study was trained by a faculty member of the department of psychiatry, AIIMS Bhubaneswar about various components of PHQ-9 and GAD-7 questionnaires before administering them to study participants. Pre-testing of the interview schedule was done among 10 individuals (in a site different from the study area), and changes were made accordingly.

Data collection

Data collection was done in the months of May and June, 2017, after obtaining written informed consent from the participants. Those who were able to read were given 20 minutes to fill out the questionnaires in an isolated room. For the rest, questions were read out verbally in an isolated room with no third person present. For cases, details of the disease were taken as per the diagnosis of a dermatologist. 

Ethical consideration

The study was started after taking ethical clearance from the Institutional Ethics Committee (IEC) of AIIMS Bhubaneswar. All the details regarding patient identification and clinical condition were kept confidential.

Statistical analysis

Descriptive statistics were presented as proportion and mean, as applicable. The socio-demographic characteristics of patients and controls were compared. Prevalence of depression and anxiety and mean score of PHQ-9 and GAD-7 were calculated and compared using the Chi-square test and Student’s t-test, respectively. Results of the bivariate analysis were reported as odds ratio (95% confidence Interval). A p-value of <0.05 was considered significant. The mean score of HRQoL was calculated using WHOQoL-BREF and was compared using Student’s t-test.

## Results

Out of the 101 patients in this study, 68 (67.3%) were males. Approximately half (n=47, 46%) of the patients were in the age group of 18-30 years. The mean age (±SD) was 36.45 (16.2) years. The controls also had the same distribution as they were matched by age and sex. Almost one-fourth (n=25, 25%) of the patients were smoking/consuming tobacco and drinking alcohol. Chronic disorders other than skin disorders (diabetes, hypertension, etc.) were present in 11 (10.89%) patients (Table 1).

**Table 1:** Comparison of cases and controls with respect to socio-demographic characteristics

Variable			Category		Cases	Control
Sex			Male		68 (67.32%)	68 (67.32%)
			Female		33 (32.67%)	33 (32.67%)
Mean age (±SD)					36.45 (16.2)	36.48 (16.3)
Age distribution		18-30 years			47 (46.53%)	47 (46.53%)
		31-40 years			19 (18.81%)	19 (18.81%)
		41-60 years			24 (23.76%)	24 (23.76%)
		>60 years			11 (10.89%)	11 (10.89%)
Illiterate					10 (9.9%)	12 (11.88%)
Smoking or consuming tobacco					25 (24.75%)	16 (15.84%)
Drinking alcohol					26 (25.74%)	8 (7.92%)
Area of stay				Rural	54 (53.46%)	54 (53.46%)
				Urban	47 (46.53%)	47 (46.53%)
Marital status				Ever married	58 (57.42%)	57 (56.43%)
				Never married	43 (42.57%)	44 (43.56%)
Chronic diseases (%)					13 (12.87%)	11 (10.89%)
Mean years of schooling					10.23	11.57
Schooling years	0 - <5 years				19 (18.81%)	21 (20.79%)
	5 - <8 years				22 (21.78%)	21 (20.79%)
	8 - <12 years				20 (19.8%)	12 (11.88%)
	≥ 12 years				40 (39.6%)	47 (46.53%)
Occupation	Unskilled/ semi-skilled				38 (37.62%)	42 (41.58%)
	Skilled				21 (20.79%)	22 (21.78%)
	Student/unemployed				31 (30.69%)	27 (26.73%)
	Homemaker				11 (10.89%)	10 (9.9%)

Most common diseases in patients were tinea (22.8%), acne (18.8%), vitiligo (14.8%), endogenous dermatitis (13.9%), psoriasis (9.9%), vasculitic ulcer (4.9%), pemphigus (2.9%), pyoderma gangrenosum (2%) and androgenic alopecia (2%). Others like hyperkeratotic eczema, chronic superficial folliculitis, sub-acute lupus erythematosus, Reiter’s syndrome, Hansen disease, epidermolytic hyperkeratosis, alopecia areata, and ichthyosis contributed to <1%.

Most (76.24%) of the patients were taking some form of treatment at the time of the study. The disease duration was of three to six months in 32%, six months to three years in 35%, and more than three years in 33% of the patients, with a mean duration of 43.9 (± 64.0) months. Three-fourths (74%) of the patients had visible skin lesions on the exposed body parts (head, neck, hand, foot).

Disease severity of each disease was calculated using various disease severity indices as detailed in the methodology. The disease severity was mild in 45 (45%) patients and moderate to severe in 56 (56.56%) patients.

Out of the 101 patients, about half (49.5%) had clinically significant (large to extremely large) impairment of their dermatological quality of life (QoL). The mean DLQI score was 10.75 (± 6.75).

Clinically significant (moderate to severe) depression was found in 45.5% of the patients (Table 2).

**Table 2:** Distribution of patients according to the severity of depression PHQ-9 - Patient Health Questionnaire-9

Category (score)	Frequency/mean	Percentage (%)
None/mild depression (<10)	55	54.45
Minimal or none (0-4)	24	23.76
Mild (5-9)	31	30.69
Moderate to severe (≥10)	46	45.54
Moderate (10-14)	27	26.73
Moderately severe (15-19)	9	8.91
Severe (20-27)	10	9.9
Mean (±SD) PHQ-9 Score	9.43 (±6.24)	

Clinically significant (moderate to severe) anxiety was found in 41.58% of the patients (Table 3).

**Table 3:** Distribution of patients according to the severity of anxiety GAD-7 - Generalized Anxiety Disorder-7

Category (score)	Frequency/Mean	Percentage
No/mild anxiety (<10)	59	58.42
No anxiety (0-4)	35	34.65
Mild (5-9)	24	23.76
Moderate to severe (≥10)	42	41.58
Moderate (10-14)	34	33.66
Severe (15-21)	8	7.92
Mean (±SD) GAD-7 score	7.64 (±5.1)	

The prevalence of depression and anxiety was significantly higher in cases as compared to the controls. Both mean PHQ-9 and mean GAD-7 scores of patients were significantly higher (p-value<0.001) than that of the controls (Table 4).

**Table 4:** Comparison of depression and anxiety among cases and controls PHQ-9 - Patient Health Questionnaire-9, GAD-7 - Generalized Anxiety Disorder-7

Category	Characteristic	Cases, controls	OR (95% CI), p-value
Depression (PHQ-9)	Prevalence	45.54%, 16.83%	4.13 (2.06-8.45), p-value <0.001
	Mean score	9.43(±6.24), 5.37(±4.52)	95% CI: 2.55-5.57, p-value <0.001
Anxiety (GAD-7)	Prevalence	41.58%, 13.86%	4.42 (2.13-9.51), p-value <0.001
	Mean score	7.64(±5.1), 4.6(±4.05)	95%CI: 1.74-4.23, p-value <0.001

**Figure 1:**
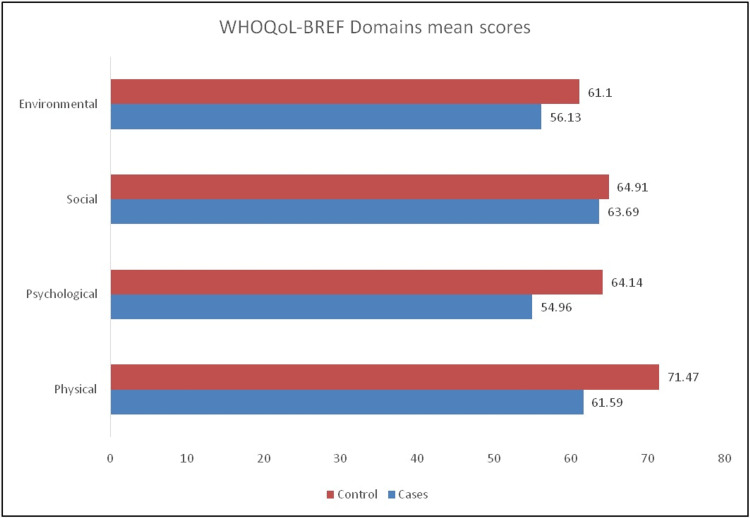
Comparison of WHOQoL-BREF mean scores among cases and controls WHOQoL-BREF - World Health Organization Quality-of-Life Scale

The mean HRQoL (using WHOQoL-BREF converted into 0-100 scale) of patients is given in Figure 1. Mean scores were highest for the physical domain and least for the environmental domain. No statistically significant difference in mean HRQoL scores was found between the cases and controls.

Univariate analysis was done to assess the effect of patient-related factors (gender, education, marital status, occupation, and presence of skin disorders on exposed body parts, etc.) on psychological distress and quality of life. No patient-related factors were significantly associated with DLQI, anxiety, or HRQoL. Only marital status was positively associated with depression.

## Discussion

In this study, we assessed and compared the psychological distress and QoL among patients presenting with chronic skin disorders and controls without any apparent skin disorder.

Out of 101 patients in this study, 67% were males, and 33% were females. A similar study done in Kerala [13] had 43.8% females, and a study in Saudi Arabia [14] had 63.3% females. These differences might be because of the study setting, awareness, and health-seeking behavior among females in the respective places.

The most common diseases in the present study were tinea (22.7%), acne (18.8%), and vitiligo (14.85%). The prevalence of various skin disorders is similar to studies done in South India [15] and the United Kingdom [16]. In the present study, 76.24% of the patients were taking treatment at the time of study, and 74% had visible skin lesions on the exposed body parts. The findings were similar to a study in Saudi Arabia where 73.9% of patients were taking medications, and 66.1% of patients had lesions in exposed body parts [14].

The mean DLQI score in the present study was 10.75 (± 6.75), higher than a study done by Singh et al., i.e., 6.85 (±6.22) [17]. The difference in scores might be attributed to the fact that our study included patients with any chronic skin disorder, whereas only patients diagnosed with psoriasis were included in the study by Singh et al. [17]. The variation might also be attributed to other diseases and cultural factors. However, in a study done in Korea, DLQI was similar to the present study, i.e., 10.7 (± 7.9) [18]. In our study, males were found to have more impaired dermatological QoL as compared to that of females, but the results were not statistically significant. The finding was contrary in some studies [14, 18, 19], and some studies did not find any association between gender and QoL [13, 20]. Poorer DLQI scores were found in patients with skin disorders in exposed body parts. A study by Abolfotouh et al. in Saudi Arabia also found significantly impaired QoL in patients with lesions in the head and face [14]. This may seem obvious as the exposed skin lesions might make the person feel more uncomfortable during social interactions.

In the present study, 45.54% and 41.58% of patients had clinically significant (moderate to severe) depression and anxiety, respectively. The mean PHQ-9 and GAD-7 scores were 9.43 (±6.24) and 7.64 (±5.1), respectively. A study done in South India among psoriasis patients found the prevalence of depression and anxiety as 78.9% and 76.7%, respectively [21]. But a study in Cameroon found the prevalence of depression and anxiety as 6.1% and 7.7%, respectively, in acne patients [22]. As the prevalence of depression and anxiety has a large variation with respect to different diseases, it seems plausible that we got some intermediate values as our study was not disease-specific, and we included various chronic skin disorders with a duration of more than three months.

Depression and anxiety scores were found to be significantly high in unmarried persons and more in unemployed people. This might be because in the Indian scenario, both the categories consist more of a younger age group of people, and they are more likely to have depression and anxiety [23, 24]. That's because chronic skin disease affects their QoL, future career options, social life, etc. Females had a lower prevalence of depression and anxiety as compared to males, but the results were not statistically significant. This finding is in contradiction with many other global studies [19, 23, 24]. But a study by Kumar et al. in North India found no relation between gender and psychosocial distress [19]. Depression and anxiety were higher in patients with skin lesions involving exposed body parts like the head, neck, hand, and foot. Similar results were found in some other studies [14, 24].

Individual controls were taken for each patient, matched by age, sex, and place of residence (rural/urban). The depression and anxiety scores were found to be significantly higher in the patients (45.54% and 41.58%, respectively) as compared to the controls (16.83% and 13.86%, respectively). Higher prevalence in cases as compared to controls is in accordance with a study by Golpour et al., where 45% and 18% of cases and controls had anxiety, and 67% and 12% of the cases and controls had depression, respectively [19].

In HRQoL, scores of all four domains (physical, psychological, social, and environmental) were found to be lower in the cases as compared to the controls, but no significant difference was found.

This study has assessed DLQI, psychological distress, and HRQoL in patients suffering from multiple chronic skin disorders. However, the number of patients in different disease spectra was very few and was highly variable. Since the data is from a tertiary care hospital, the results might not be generalizable. 

## Conclusions

Depression and anxiety were found to be significantly higher in the patients with chronic skin disorders as compared to the controls without apparent skin disorders. Also, in comparison to the controls, patients had higher impairment of dermatological quality of life and lower mean health-related quality of life scores. Screening of anxiety, depression, and quality of life should be done in the dermatology OPD for chronic skin disorder patients. Tailored psychiatric consultation and appropriate counseling should be offered to those in need.
